# Fast hyperbaric decompression after heliox saturation altered the brain proteome in rats

**DOI:** 10.1371/journal.pone.0185765

**Published:** 2017-10-04

**Authors:** Alvhild Alette Bjørkum, Eystein Oveland, Linda Stuhr, Marianne Bjordal Havnes, Frode Berven, Marit Grønning, Arvid Hope

**Affiliations:** 1 Department of Biomedical Laboratory Sciences and Chemical Engineering, Western Norway University of Applied Sciences, Bergen, Norway; 2 Proteomics Unit at the University of Bergen, Department of Biomedicine, Faculty of Medicine and Dentistry, University of Bergen, Bergen, Norway; 3 Department of Clinical Medicine, Faculty of Medicine and Dentistry, University of Bergen, Norway; 4 Department of Biomedicine, Faculty of Medicine and Dentistry, University of Bergen, Bergen, Norway; 5 Department of Circulation and Medical Imaging, Faculty of Medicine and Health Sciences, Norwegian University of Science and Technology, Trondheim, Norway; 6 Department of Occupational Medicine, Haukeland University Hospital, Bergen, Norway; 7 NUI AS, Bergen, Norway; McGill University, CANADA

## Abstract

Better understanding of the physiological mechanisms and neurological symptoms involved in the development of decompression sickness could contribute to improvements of diving procedures. The main objective of the present study was to determine effects on the brain proteome of fast decompression (1 bar/20 s) compared to controls (1 bar/10 min) after heliox saturation diving, using rats in a model system. The protein S100B, considered a biomarker for brain injury, was not significantly different in serum samples from one week before, immediately after, and one week after the dive. Alterations in the rat brain proteome due to fast decompression were investigated using both iontrap and orbitrap LC-MS, and 967 and 1062 proteins were quantified, respectively. Based on the significantly regulated proteins in the iontrap (56) and orbitrap (128) datasets, the networks “synaptic vesicle fusion and recycling in nerve terminals” and “translation initiation” were significantly enriched in a system biological database analysis (Metacore). Ribosomal proteins (RLA2, RS10) and the proteins hippocalcin-like protein 4 and proteasome subunit beta type-7 were significantly upregulated in both datasets. The heat shock protein 105 kDa, Rho-associated protein kinase 2 and Dynamin-1 were significantly downregulated in both datasets. Another main effect of hyperbaric fast decompression in our experiment is inhibition of endocytosis and stimulation of exocytosis of vesicles in the presynaptic nerve terminal. In addition, fast decompression affected several proteins taking parts in these two main mechanisms of synaptic strength, especially alteration in CDK5/calcineurin are associated with a broad range of neurological disorders. In summary, fast decompression after heliox saturation affected the brain proteome in a rat model for diving, potentially disturbing protein homeostasis, e.g. in synaptic vesicles, and destabilizing cytoskeletal components. Data are available via ProteomeXchange with identifier PXD006349

## Introduction

For many years it has been discussed whether professional divers are at risk of long-term health effects caused by their diving career [1,2,3]. Among the possible causes and explanations for CNS changes are the occurrence of gas bubbles and decompression sickness (DCS) [2]. Studies on a possible relationship between decompression sickness and lesions in the brain and spinal cord determined by magnetic resonance imaging (MRI) have shown divergent results [4,5,6,7,8]. Increased prevalence of white matter changes is demonstrated in healthy individuals/divers without neurological decompression sickness [9,10]. This also applies for recreational divers, and a minor decrease in neuro-cognitive performance has been demonstrated in some individuals [11,12]. Moen et al. (2010) observed regional functional abnormalities in former saturation divers from diffusion- and perfusion-weighted MRI [13]. They found that the perfusion deficits in the watershed areas were consistent with arterial microemboli or some general dysfunction of cerebral microvascular function. The mean transition time was reduced which could be due to reduced flexibility of the microvascular system or reduced capillary complexity which they assumed to be long-term clinical symptoms reported by professional divers.

A set of biomarkers available for monitoring status of the cellular physiological mechanisms involved in the development of DCS and neurological symptoms could contribute to improvements of diving procedures. Previous experiments in our laboratory has shown that decompression from heliox saturation at a rate of 1 bar/20 s (fast), resulted in DCS symptoms in approximately 50% of the animals, whereas the slow rate of 1 bar/10 min did not result in DCS or venous gas bubbles [14,15]. Structural MRI did not reveal morphological changes in CNS in rats with severe bubble formation and DCS symptoms after air and heliox dives [14,16]. However, from functional MRI, Havnes et al. (2013) concluded that circulatory changes might occur in the brain during the acute phase [16]. Increased serum levels of the protein biomarker S100B, predominantly found in brain glial cells, have been associated with brain injury [17]. However, physiological and methodological aspects should be considered regarding measurements of serum S100B [18]. Havnes et al. (2010) observed higher levels of serum S100B in rats with high compared to rats with low bubble grades after air dives [19]. Furthermore, cellular effects and potentially persistent effects on transcriptomic/gen-expression have been observed after scuba diving [20].

The main objective of the present study were to investigate changes in serum levels of the biomarker S100B and changes in protein levels (proteomic analysis) in the brain during fast (1 bar/20 s) decompression compared to controls (1 bar/10min) after heliox saturation diving.

## Methods

### Rats, experimental procedures and sample preparation

Two groups of female Wistar rats (Charles Rivers Laboratories, USA) weighing approximately 250 g were studied. The rats were kept under 12:12-h light-dark cycles in cages with 4 animals together in a mobile cabinet with built-in ventilation and air filtration on the air inlet as well as on the exhaust. All animals had free access to drinking water and standard rat chow throughout the exposure period. All protocols were performed in accordance with the Norwegian Regulation on Animal Experimentation and approved by the Norwegian Animal Research Authority (S-2007/5669, id 195).

All rats were trained to walk on a treadmill (Treadmill Simplex II, Columbus Instruments, Columbus, Ohio, USA) for later determination of signs and symptoms of DCS. This training took part during the one-week acclimation period before the hyperbaric exposure. The animals were weighed every morning. On the day of the hyperbaric exposure, 2 or 3 rats were placed in the 130 L pressure chamber. The chamber was flushed with heliox for 20 minutes to remove nitrogen [15] and pressurized with heliox (80:20) to 2.5 bar, giving a pO_2_ of approximately 50 kPa, followed by pure helium infusion to obtain the final pressure of 5 bars. The chamber temperature was kept at approximately 26°C during the 5 bar exposure. Three hours later the animals were decompressed at rates of 1 bar/20 s (FD, fast decompression) or 1 bar/10 min (SD, slow decompression) (Table 1). When pO_2_ reached 16 kPa during the linear decompression in group SD (control group), pure oxygen was injected to reach a pO_2_ of about 25 kPa before decompression was continued to surface pressure. In the FD group, where the total decompression time was less than two minutes, the pO_2_ was not adjusted for.

**Table 1:** An overview of number of rats, saturation time and decompression rates.

Group	Total no. of rats	Saturation time, hours	Decomp. rate	Brain samples for prot. exp., no. of rats	Blood samples for S100B, no. of rats		
					1 week before dive	2–3 hours after dive	1 week after dive
**FD**	21	3	1 bar/20 s	10	6	8	7
**SD**	11	3	1 bar/10 min	10			11

FD = fast decompression, SD = slow decompression.

Immediately after surfacing, the rats were investigated for DCS symptoms, initially in the cage, and approximately 5–10 min afterwards while walking on the treadmill. Observation on and recordings of pulmonary and neurological symptoms, such as heavy and fast breathing, chokes, unconsciousness, and complete or partial paralysis in one or more legs, were performed. All animals survived and none was treated for DCS symptoms by recompression.

One week after the dive the animals were euthanized with CO_2_, the rats were intracardially perfused with sterile saline (remove traces of blood) and the brain dissected out before snap frozen in liquid nitrogen, and stored at -80°C until further protein analysis.

### S100B serum analysis

Approximately 1 ml venous blood samples were obtained from the tail vein during gas anaesthesia (Isoflurane/N_2_O) by sampling one week before, 2–3 hours after, and one week after the dive. Thereafter, protein S100B analyses were performed by enzyme-linked immunosorbent assay (ELISA) using a commercial kit (BioVendor-Laboratorní medicína, Brno-Modice, Czech Republic).

### Sample preparation for mass spectrometry analyses

The protein level profile in brain tissue samples from the SD group (n = 10) and the FD group (n = 10) were compared using quantitative proteomics analyses. From each animal one of the cerebral hemispheres (approximately 0.7 g), excluding both the frontal lobes and the cerebellum, was cut in small pieces with scalpel. The pieces were mixed with fresh ice-cold homogenisation buffer (50 mM Hepes, pH 7.4, containing 100 mM KCl and proteases inhibitors cocktail from Roche Diagnostics Gmbh) at the ratio ¼ (w/v)- add 4 ml to 1 gr- and homogenised in Potter-Elvehjem. The homogenate was centrifuged for 15 min at 20 000 g at 4°C and the supernatant was collected, aliquoted and immediately frozen at -80°C.

### Iontrap mass spectrometry and Spectrum Mill

Protein samples were fractionated in 12 fractions by 1D SDS-PAGE electrophoresis prior to iontrap LC-MS. Before application on the gel, one aliquot of proteins (ca. 70 μg) each was reduced and alkylated as following. Samples were reduced with 50mM dithiothreitol and incubated in loading buffer (Laemmli sample buffer) for 10 minutes at 70°C. Reduced proteins were alkylated by adding 1/10 volume of 200 mg/ml iodoacetamide and incubated for 30 min at room temperature in the dark. Electrophoresis was then performed on Invitrogen NUPAGE 4–12% Bis-Tris gel (1.0 mm tick) using MOPS buffer at a voltage of 185 V and current 200 mA for about 1 hour. Proteins were stained with Coomassie Blue to get a profile of proteins separation. Two gels, each containing 5 samples derived from controls and 5 samples derived from treated animals, were run in parallel resulting in identical stained protein profile for the 20 lanes. Each gel lane was cut in 12 pieces of equal size. Each large piece was placed in a well of a 96 wells plate and further nicely cut with a scalpel, not crushed, into 4–6 smaller pieces. The pieces were destained and treated separately with trypsin (25 μl of 10 ng trypsin /μl Triethylammonium bicarbonate buffer with concentration 5 mM at 37°C for 4 hours. After washing and extensive extraction of peptides, including at last an extraction from with 100% acetonitrile (ACN), the collected extracts containing the peptides were lyophilized and resuspended in 10 μl formic acid 5% (v/v).

Approximately 1μg of peptides were applied and analysed on an Iontrap mass spectrometer (LC/MSD Trap XCT plus from Agilent Technologies) resulting in 240 LC-MS raw data files. The raw data files were analysed in Spectrum Mill using the workflow and settings as previously described [21,22] and the SwissProt Rat database. The identified proteins were summarized and exported for quantitative analysis using the average peptide spectrum intensity.

### Orbitrap mass spectrometry and Progenesis LCMS

Protein samples were trypsinized in solution and approximately 1μg of peptides were subjected to LC-MS analysis using 88 min runs with a biphasic acetonitrile gradient on an Ultimate NCS-3500RS HPLC and a nanoViper column coupled to an LTQ-Orbitrap Velos Pro as previously described [23]. The top 7 peaks from the MS scan were fragmented in the MSMS analysis; minimum signal counts required were 1000.

The orbitrap raw files were analyzed in Progenesis LC-MS^®^ v2.6 (Nonlinear Dynamics Ltd). The chromatographic features were automatically aligned (>96.8% alignment score), and only features with charges between +2 to +6 with associated MS/MS spectra were accepted for export (mgf file) for identification using X!Tandem and OMSSA in SerachGUI [24] with the SwissProt Rat canonical database. The search criteria were: trypsin with one miss-cleavages accepted, fixed carbamidomethylation on cystein, variable oxidation on methionine, precursor mass tolerance of 10 ppm and fragment mass tolerance of 0.7. The search result and associated spectra were combined and assigned to proteins in PeptideShaker [25] at 1% FDR and exported to Progenesis (all validated Peptide Spectrum Matches) and assigned to the features. The protein abundances reported were based on the sum of the normalized abundance of the quantified unique peptides.

### Analyses of the proteomics data

Metacore v6.26 (Thomson Reuters) was used to analyse the significantly regulated proteins in the FD group compared to the SD group resulting from the iontrap and orbitrap analyses. String v10.0 [26] was used to analyze the significantly regulated proteins with more than >20% regulation for potential protein-protein interactions and GO enrichments. Venny 2.1.0 was used to compare data sets (http://bioinfogp.cnb.csic.es/tools/venny/index.html). Perseus v1.5.3.2 [27] was used to generate the unsupervised clustering heatmap with dendrograms and PCA plots.

### Publication of LC-MS data in PRIDE

The proteomics raw files and search/quantification results from the orbitrap analysis have been deposited to the ProteomeXchange Consortium [28] via the PRIDE partner repository [29] with the dataset identifier PXD006349.

## Results

In the present experiments 12 of 21 rats in the fast decompression rate group (FD, 1 bar/20 s) got DCS symptoms and, as expected, such symptoms were not observed in any rats from the SD group (SD, 1 bar/10 min).

### Protein S100B levels in serum

**Fig 1:**
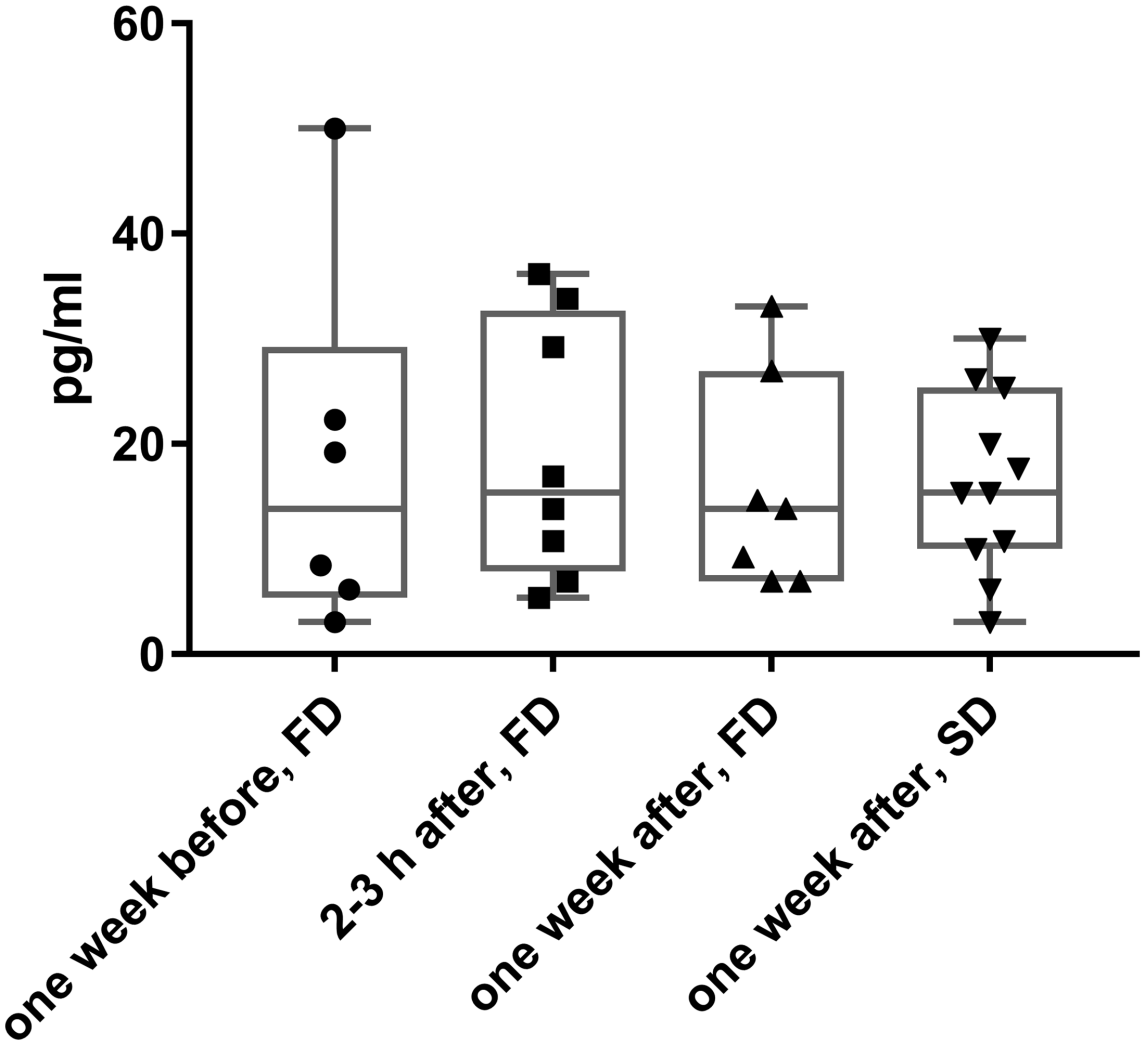
Protein S100B levels in serum after hyperbaric exposure. Box plots of serum S100B concentration one week before, 2–3 hours after, and one week after the dive in the fast decompression rate group (FD, 1 bar/20 s), and one week after the dive in the slow decompression rate group (SD, 1 bar/10 min). The plot shows the 25th and 75th percentiles with median and bars at maximum and minimum values.

Elevated levels of S100B has been evaluated as a biomarker for brain injury [17,19]. ELISA analyses of serum S100B showed no significant differences in levels when comparing the FD groups from one week before, immediately after, and one week after the dive to the SD group one week after the dive (Fig 1). Thus, indication of brain injury was not detected in the FD group rats at the experimental conditions and timepoints used based on the serum S100B analysis.

### The rat brain proteome after hyperbaric exposure with fast compared to slow decompression

**Fig 2:**
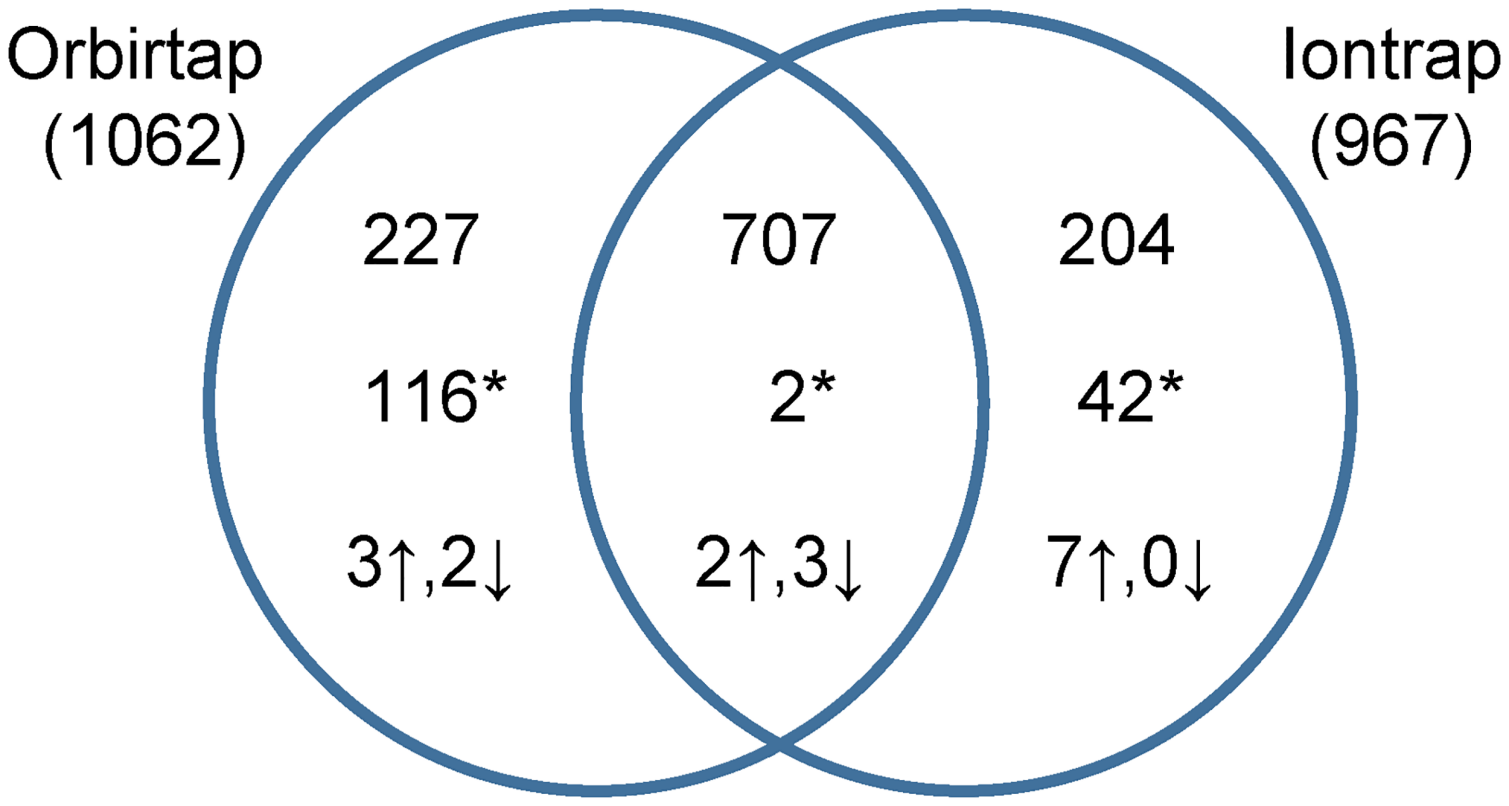
Proteins quantified using iontrap and orbitrap LC-MS. The total number of proteins quantified using the different methods are presented in parenthesis outside the diagram. The overlapping identifications are shown within the sections and the intersection. The numbers at the top in the diagram show number of proteins without significant regulation. The significantly regulated proteins due to fast decompression, according to two-sided t-tests, are denoted with an asterisk (*, p<0.05). The significantly regulated proteins which in addition passed the filtering criteria (>20% regulation and significant in both datasets) are illustrated with arrows.

The quantification of the proteins detected from rat brains, fast decompression rate group (FD, n = 10) versus slow decompression rate group (SD, n = 10), was done by using two different label-free mass spectrometry based proteomic analyses. First, the brain lysate samples were fractionated and subjected to iontrap LC-MS followed by MS2 spectral intensity analysis in Spectrum Mill, which resulted in quantification of 967 proteins (Fig 2). Second, the samples were also subjected to orbitrap LC-MS followed by MS1 AUC analysis in Progenesis LCMS, resulting in quantification of 1062 proteins. Statistical analyses revealed 128 significantly regulated proteins in the orbitrap dataset and 56 significantly regulated proteins in the iontrap dataset, respectively (S1 and S2 Tables). These proteins were investigated further using clustering analyses and the system biology database MetaCore. Next, a filter criterion of >20% regulation was applied to reveal the most regulated proteins, resulting in 19 candidates. The 19 candidates were compared to the results from the system biology analyses in order to reveal the processes with most significant biological relevance.

### Clustering analyses of the proteomics data sets

**Fig 3:**
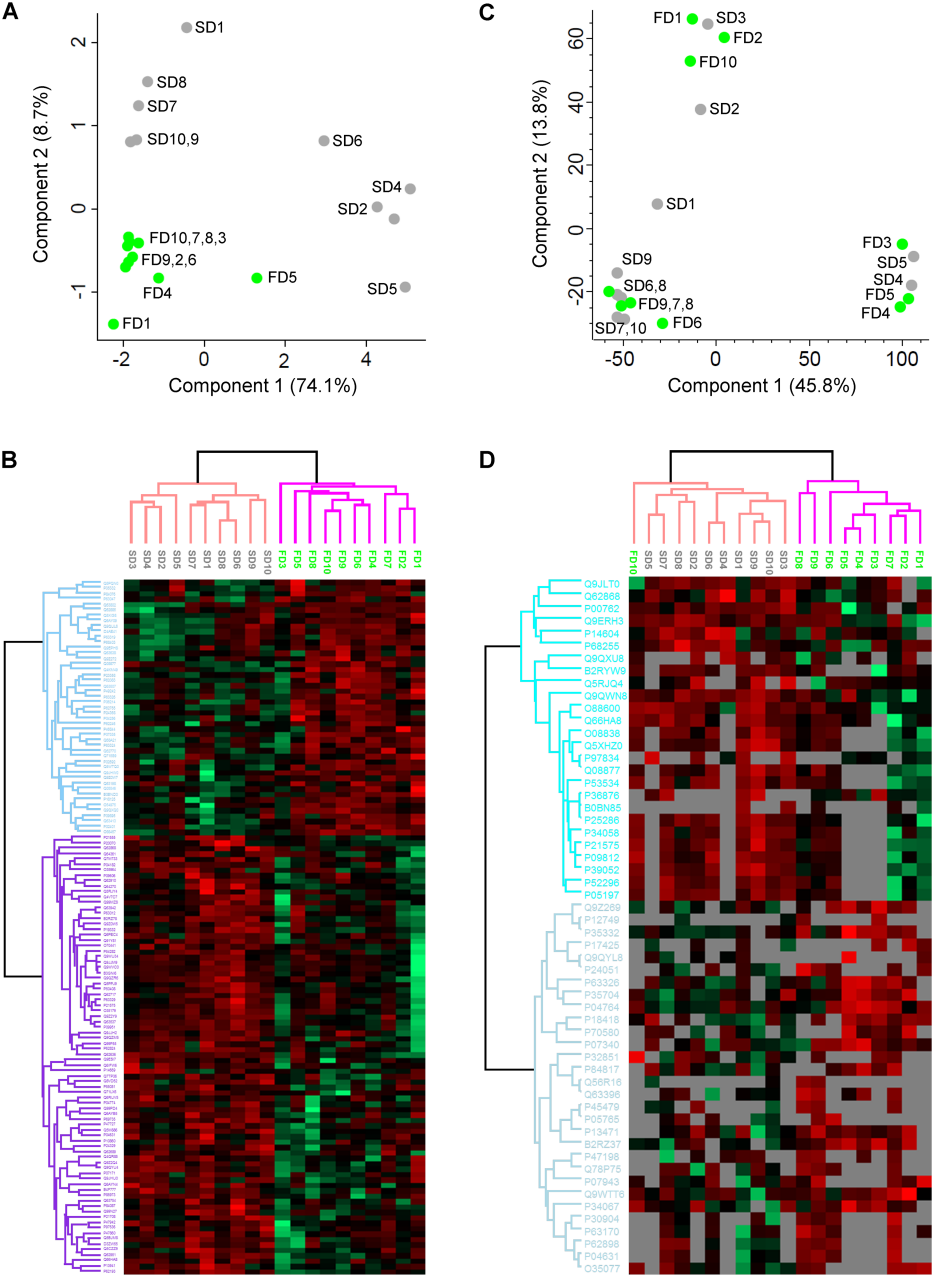
Clustering analyses of all significantly regulated proteins. The normalized protein quantification values, from significantly regulated proteins in the orbitrap analysis (A, B) and iontrap analysis (C, D) were imported into Perseus. Log2 transformed data were analysed with principal component analysis for the orbitrap dataset (A) and the iontrap dataset (C), NaN values were replaced with 0 for the latter. The log2 data was then z-normalized by row and unsupervised hierarchically clustered (distance: Spearman, linkage: average) for the orbitrap dataset (B) and the iontrap dataset (D), including NaN illustrated as grey cells for the latter. Green cells represent downregulated proteins and red cells upregulated proteins. FD, fast decompression rate; SD, slow decompression rate.

Orbitrap LC-MS followed by Progenesis LCMS analysis is considered to be among the most developed label-free quantification strategies, giving quantitative values for all replicates even if the peptide was not identified by MS/MS in each of the replicates. This strategy enabled powerful hierarchical clustering and PCA analysis with valid values for all 128 significantly regulated proteins in all replicates for the Orbitrap dataset. The significantly regulated proteins resulted in two clusters containing only proteins from the FD and SD-groups/animals, respectively, both in PCA and in a hierarchical clustering analysis (Fig 3A and 3B).

A number of 56 significantly regulated proteins (p>0.05) from the iontrap Spectrum Mill data contained several missing values. These were replaced by zero for the PCA analysis, resulting in an incomplete separation of the two groups based on the significantly regulated proteins (Fig 3C). Imputation of the missing values with NaN (Not a Number) for the significantly regulated proteins in the iontrap dataset resulted in separation of the FD and SD groups using hierarchical clustering, except for the sample FD10 which clustered together with the SD group (Fig 3D). Thus, both the significantly regulated proteins from the iontrap and the orbitrap analyses were investigated further, as potential biomarker candidates for fast decompression rate after hyperbaric exposure.

### System biology analyses of all significantly regulated proteins

**Fig 4:**
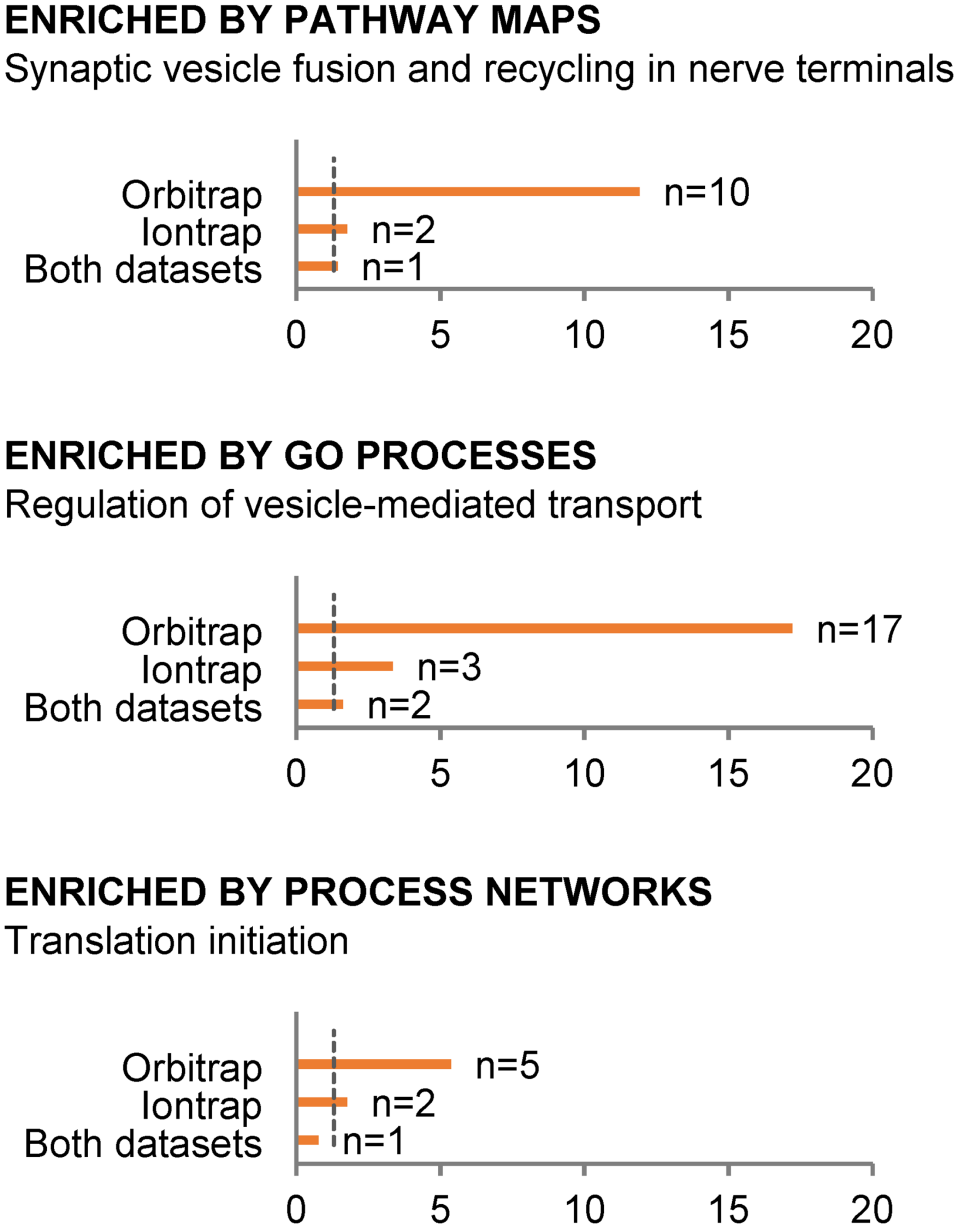
Enriched ontologies and networks in Metacore. The number of proteins (n) in each enriched terminology is shown. The -log2 (p-value) is plotted and the p = 0.05 illustrated with a stippled line.

**Fig 5:**
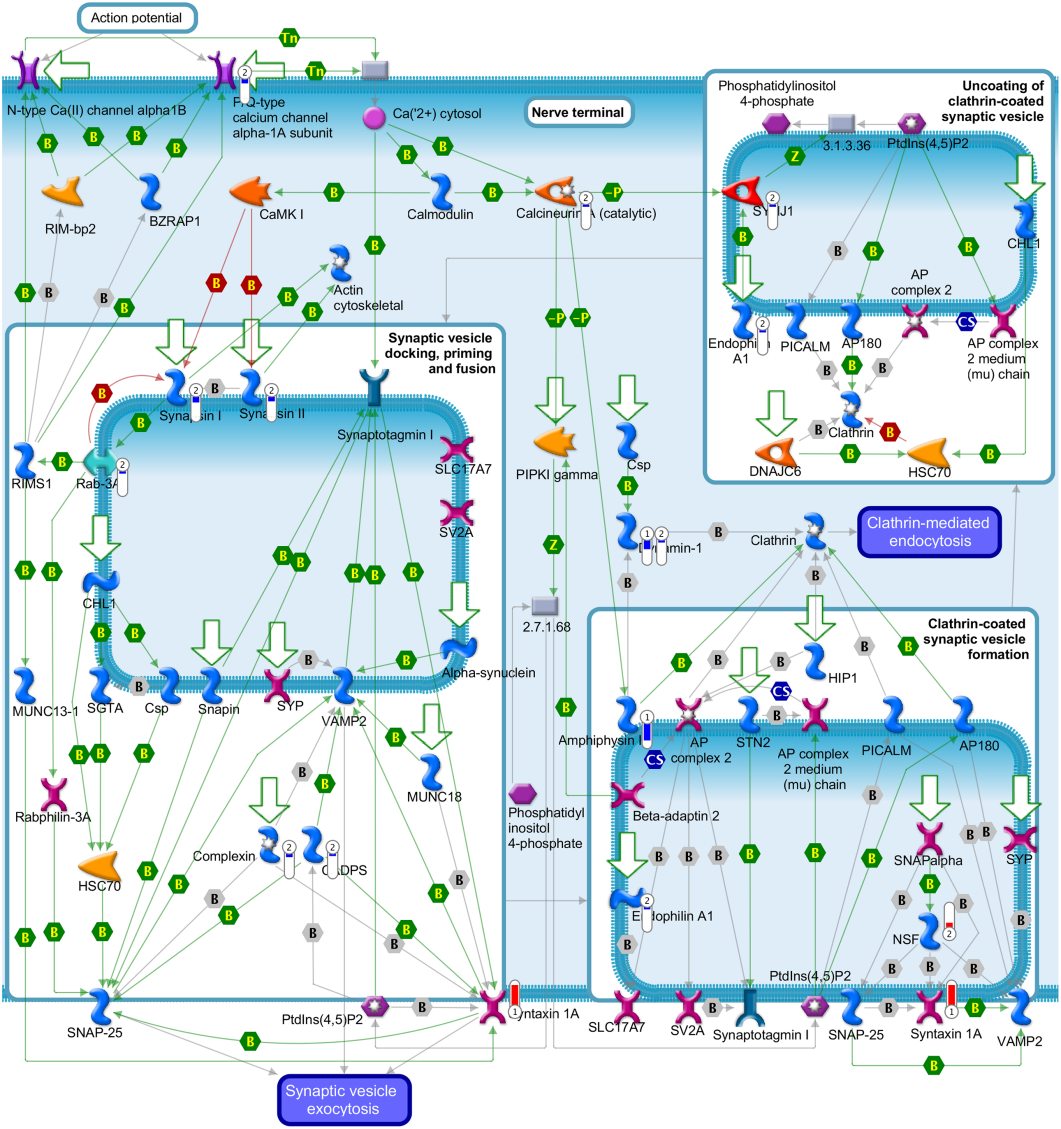
Synaptic vesicle fusion and recycling in nerve terminals. The thermometers indicates upregulation (red) or downregulation (blue). Pathway symbol explanations: https://ftp.genego.com/files/A4_MetaCore_qrg_en.pdf.

The proteins significantly regulated due to the fast decompression from the iontrap (56) and orbitrap (128) datasets were analysed in Metacore (Thomson Reuters). The most statistically significant enriched ontologies and networks based on these proteins are presented in Fig 4. These include the gene ontology term “regulation of vesicle-mediated transport” and the subgroup of “neurophysiological process” named “synaptic vesicle fusion and recycling in nerve terminals” (Fig 5). Also the process network “translation initiation” was significantly enriched (S1 Fig). All the regulated proteins were submitted to the “build network analysis” in Metacore and interactions visualized (S2 Fig). The “signal transduction_ESR1-nuclear pathway” was postulated as regulated; in that network the downregulation of HSP70 might result in the upregulation of DLC1, the gene name for dynein light chain 1, cytoplasmic (DYL1).

### Potential protein biomarker candidates for hyperbaric exposure

The quantified proteins which were regulated >20% due to the fast decompression in both LC-MS methods (Table 2), and only detected in either the iontrap or the orbitrap LC-MS analysis and regulated >20% (Table 3) were investigated further.

**Table 2:** Proteins regulated in the brain of diving rats in both orbitrap and Iontrap analyses.

Description	Protein_Entry	Orbitrap				Iontrap			
		Pept	Spec	FD/SD	p	Pept	Spec	FD/SD	p
Hippocalcin-like protein 4	HPCL4_P35332	1	25	1.22	0.019	10	82	2.30	0.015
40S ribosomal protein S10	RS10_P63326	2	23	1.10	0.008	4	35	1.58	0.009
60S acidic ribosomal protein P2	RLA2_P02401	1	17	1.21	0.000	1	1	§	§§
Proteasome subunit beta type-7	PSB7_Q9JHW0	1	2	1.30	0.019	4	6	1.43	§§
Heat shock protein 105 kDa	HS105_Q66HA8	17	232	0.95	0.012	19	187	0.69	0.010
Rho-associated protein kinase 2	ROCK2_Q62868	3	4	0.93	0.024	29	149	0.68	0.032
Dynamin-1	DYN1_P21575	34	839	0.95	0.033	60	911	0.76	0.020

The protein candidates listed were >20% regulated between the fast decompression (FD) group and the slow decompression (SD) group in at least one of the quantitative proteomics analyses. Abbreviations: Pept, Peptide number used for quantification; Spec, spectra number used for identification; p, p-value calculated using two-sided T-tests expecting equal variance; § detected in the FD group only; §§, not detected in enough individuals in the SD group to calculate the p-value.

**Table 3:** Proteins only detected in either orbitrap or Iontrap and significantly regulated.

Description	Protein_Entry	Pept	Spec	FD/SD	p
Ectonucleotide pyrophosphatase/phosphodiesterase family member 6 ^a)^	ENPP6_B0BND0	2	28	1.52	0.001
RNA binding motif protein, X-linked-like-1 ^a)^	RMXL1_D4AE41	1	3	1.21	0.015
Wee1-like protein kinase ^a)^	WEE1_Q63802	1	13	1.90	0.037
Xin actin-binding repeat-containing protein 2 ^a)^	XIRP2_Q71LX6	1	2	0.79	0.006
NADH-cytochrome b5 reductase 3 ^a)^	NB5R3_P20070	1	9	0.75	0.011
40S ribosomal protein S27-like ^b)^	RS27L_P24051	2	20	1.44	0.009
Dynein light chain 1, cytoplasmic ^b)^	DYL1_P63170	7	47	1.43	0.027
60S ribosomal protein L26 ^b)^	RL26_P12749	1	4	1.24	0.028
Mitochondrial fission 1 protein ^b)^	FIS1_P84817	4	28	1.56	0.032
40S ribosomal protein S21 ^b)^	RS21_P05765	3	14	1.91	0.035
Importin subunit alpha-6 ^b)^	IMA6_Q56R16	1	4	1.49	0.040
Proteasome subunit beta type-4 ^b)^	PSB4_P34067	5	50	1.39	0.044

The protein candidates listed were >20% significantly regulated between the fast decompression (FD) group and the slow decompression (SD) group in one of the quantitative proteomics analyses and not detected in the other. The proteins regulated according to one of the methods but falsified by the other were not included. Abbreviations:

^a)^ Significantly regulated proteins in Orbitrap analysis and not detected in Iontrap;

^b)^ Significantly regulated proteins in Iontrap analysis and not detected in Orbitrap; Pept, Peptide number used for quantification; Spec, spectra number used for identification; p, p-value calculated using two-sided T-tests with equal variance.

**Fig 6:**
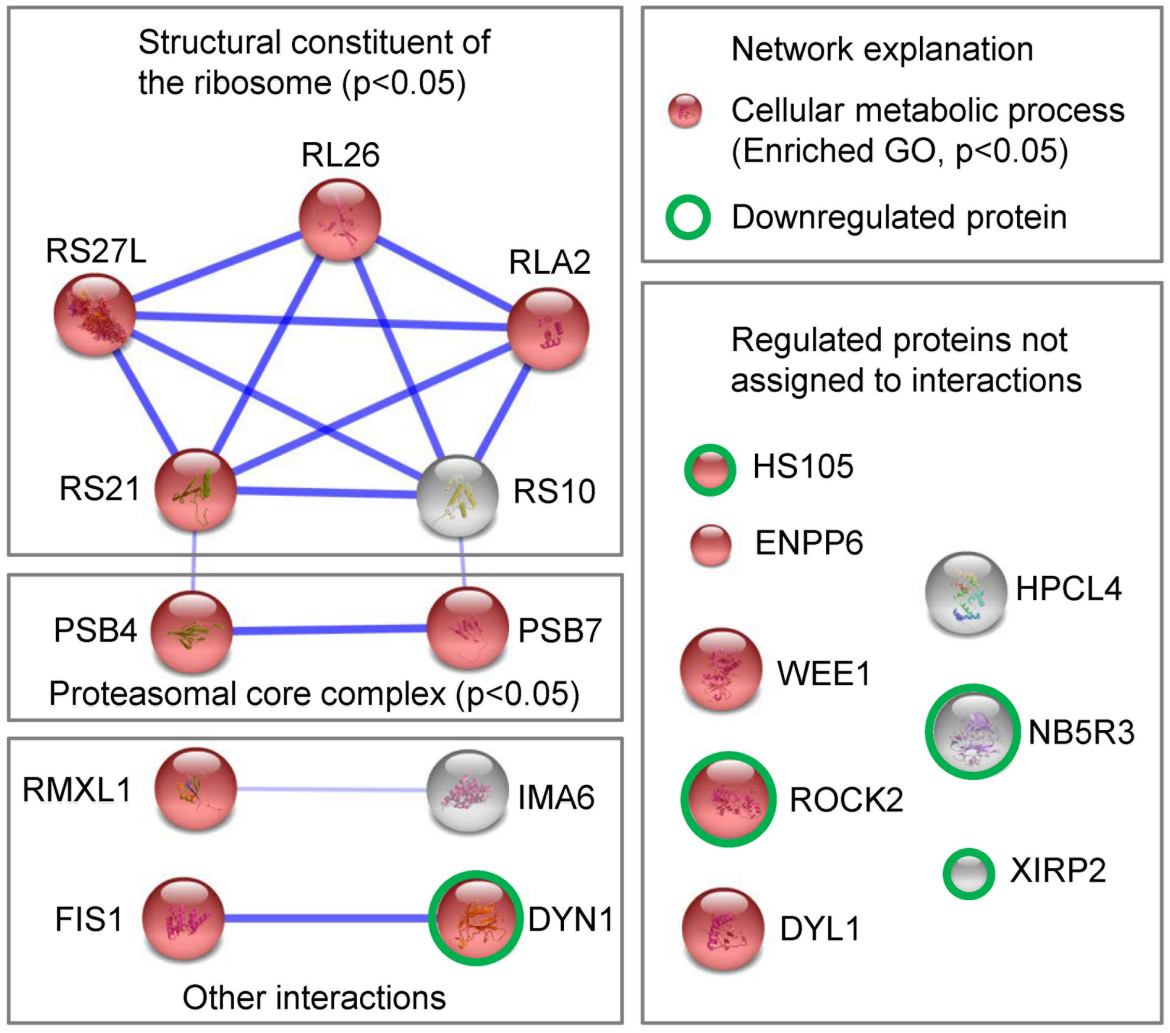
Network analysis of the >20% significantly regulated proteins. The proteins significantly regulated (>20% regulation) due to fast decompression (Tables 2 and 3) were analysed for potential protein-protein interactions and GO enrichments using String. Green circles indicate the significantly downregulated proteins in the datasets, all proteins without a green circle were significantly upregulated. Explanation of protein name abbreviations, see Table 3.

Fourteen of the nineteen >20% significantly regulated proteins in brains of rats exposed to fast decompression were associated with the biological GO term “cellular metabolic processes”, 5 with the molecular function “structural constituent of the ribosome” and 2 with “proteasomal core complex”, resulting in significant enrichment of these terms in String (Fig 6). This was in accordance with the significant enrichment in the “translation initiation” in Metacore. Regulation of a group of similar proteins in the same direction increases the likelihood that the relatively small changes in protein level detected are biologically relevant.

## Discussion

The main objective of the present study was to determine effects on the brain proteome of fast decompression (1 bar/20 s) compared to controls (1 bar/10 min) after heliox saturation diving, using rats in a model system. Upregulation of the “structural constituents of the ribosome”, three small (40S) and two large (60S) subunits in the fast decompression group indicates an increased proteins synthesis in the brain in these rats. Furthermore, two “proteosomal core complex” proteins involved in proteolysis were also upregulated in the fast decompression group. Also, importin subunit alpha-6, previously known to mediate nuclear import of the transcription factor STAT1 dimers into the nucleus, was upregulated. Taken together, these findings indicate that fast decompression induces disturbances of the protein homeostasis in the brain.

Key regulators of actin cytoskeleton and cell polarity, Rho-associated protein kinase 2, and Xin actin-binding repeat-containing protein 2, which protects actin filaments from depolymerisation, were both downregulated in the fast decompression group. Furthermore, dynamin-1, involved in producing microtubule bundles, and heat shock protein 105 kDa which prevents the aggregation of denatured proteins in cells under severe stress [30], were also downregulated. These proteins have been suggested to be involved in linking dynein to cargos and to adapter proteins, potentially playing a role in changing or maintaining the spatial distribution of cytoskeletal structures [31]. Together these findings indicate the occurrence of a destabilization of the cytoskeleton and structural components in the brain of rats exposed to fast decompression.

Several of the proteins regulated after fast decompression were are potentially affecting a diversity of cellular signalling networks. The upregulated protein hippocalcin-like protein 4 (NPV-2), a neuron-specific protein, has been postulated to be involved in calcium-dependent regulation of rhodopsin phosphorylation [32]. Wee1-like protein kinase, an important regulator of the S/G2 phase checkpoint of the cell cycle, was upregulated. Furthermore, the mitochondrial fission 1 protein which might induce cytochrome c release from mitochondria ultimately leading to apoptosis was upregulated. NADH-cytochrome b5 reductase 3 was downregulated in rats exposed to fast decompression, and defects in this protein has been associated with methemoglobinemia and tissue hypoxia [33].

The protein S100B has been considered a biomarker for brain injury [34], and also after recreational scuba diving with neurological decompression sickness [35], although the usefulness of S100B is disputed [36]. The S100B analysis showed large variations in levels and were not significantly different in serum samples from one week before, immediately after, and one week after the dive. In line with this observation, S100B also showed varying regulations in the protemics data sets (IT average FD/SD: 1.32, p = 0.019; OT average FD/SD: 0.94, p = 0.043). Whether the varying S100B levels in the brains and serum of rats are biologically relevant with respect to the fast decompression is not clear from these results.

Several of the proteins significantly regulated due to the fast decompression are of importance for regulation of vesicle-mediated transport and synaptic vesicle fusion and recycling in nerve terminals (Fig 5). The proteins play an important part in the molecular pathways and processes in the presynaptic nerve terminals, a topic recently reviewed by Fassio et al. [37]. The regulated proteins synapsin, amphiphysin and dynamins take part in the (serine/threonine) cyclin-dependent kinase 5 (CDK5)/calcineurin synaptic cellular signalling system involved in controlling the recycling pool of synaptic vesicles. These proteins are classified as main players involved in various steps in the vesicle cycle in the presynaptic nerve terminal and are among four other main specific substrates for the CDK5/calcineurin [30].

Regulation of vesicle exocytosis, trough CDK5/calcineurin phosphorylation of the protein syntaxin 1 (also known as munc 18), also takes part in the SNARE complex and vesicle fusion, hence is important for endocytosis to happen [38]. This allows the interaction of the plasma membrane protein syntaxin 1A with SNAP-25 and VAMP2 that is essentially for the formation of the SNARE complex to mediate exocytosis [38]. Therefore, the elevated level of syntaxin 1A in fast decompression rats might lead to an activation of exocytosis. Also Eftedal et al. 2013 have seen CDK5/calcineurin downregulated in the blood transcriptome of experienced divers after scuba diving [20].

The increased vesicle fusion and increased release and levels of neuronal transmitters by exocytosis suggest that the synaptic vesicle cycle is affected by hyperbaric fast decompression. This might also be one of several underlying factors for long-term neurological effects seen after repeated hyperbaric exposures [39]. However, this also seems to be in line with changed endocytosis shown through down-regulation of dynamin-1 and amphiphysin (only significant in IT) and therefore disturbed synaptic transmission in general after hyperbaric fast decompression. The endocytosis is mainly realized or effectuated via the clathrin protein [40]. Clathrin is considered to be the major protein of the polyhedral coat of vesicles and pits, and it is also known that the phosphorylation of clathrin-adaptor dynamin-1 and amphiphysin 1 and the decrease of its activity by CDK5 can inhibit the endocytosis [41]. Because of the down-regulation of the endocytosis-stimulating clathrin-adaptors, the activation of coating vesicles was decreased and destabilized by missing bindings with amphiphysin 1 and dynamin-1 with clathrin [42], it seems that hyperbaric fast decompression, as in this experiment, leads to the same effects.

Second, several Rabs (**Rab-3, Rab-3A, Rab-3C and Rab-3d**) were significantly downregulated (but not after cut-off) after hyperbaric decompression. Rab is together with Arf master regulators of membrane trafficking [37].

Taken together, the hyperbaric fast decompression in our experiment might have resulted in inhibition of endocytosis and stimulation of exocytosis of vesicles in the presynaptic nerve. Fast decompression affected several proteins taking parts in these two main mechanisms of synaptic strength, as alteration in CDK5/calcineurin shown by others, to be associated with a broad range of neurological disorders as previously reviewed [43,44].

In conclusion, fast decompression rates after heliox dives in rats did not significantly affect the serum nor brain levels of S-100B. Nevertheless, it significantly affected the brain proteome influencing synaptic vesicle fusion and recycling in nerve terminals, translation initiation, and destabilization of cytoskeletal components. These results are potentially translatable to human divers and further studies are necessary to characterize the mechanisms in more detail to make preventive actions as well as treatment strategies for divers.

## Supporting information

S1 Fig The significantly enriched Metacore network “translation initiation”. (TIF)

S2 Fig Metacore network built from all proteins regulated. (TIF)

S1 Table Proteins quantified in the orbitrap dataset with imported quantified proteins from the iontrap dataset. (XLSX)

S2 Table Proteins quantified in the iontrap dataset. (XLSX)
